# The webcam and student engagement in synchronous online learning: visually or verbally?

**DOI:** 10.1007/s10639-022-11050-3

**Published:** 2022-04-18

**Authors:** Marion Händel, Svenja Bedenlier, Bärbel Kopp, Michaela Gläser-Zikuda, Rudolf Kammerl, Albert Ziegler

**Affiliations:** 1 Department of Psychology, Friedrich-Alexander-Universität Erlangen-Nürnberg, Regensburger Str. 160, 90478 Nürnberg, Germany; 2 Department of Education, Friedrich-Alexander-Universität Erlangen-Nürnberg, Dr.-Mack-Straße 77, 90762 Fürth, Germany; 3 Department of Education, Friedrich-Alexander-Universität Erlangen-Nürnberg, Regensburger Straße 160, 90478 Nürnberg, Germany

**Keywords:** Higher education, Webcam, Online learning, Visual engagement, Verbal engagement

## Abstract

Given that video conferencing serves as a crucial means for remote teaching, the current study investigated higher education students’ (non)use of webcams and engagement in synchronous online courses. Three phases were studied: (1) A state of engagement; (2) antecedents that influence it; and (3) consequences of engagement. The cross-sectional online survey encompassed 3,610 students. Results indicated that visual and verbal engagement were only slightly related to each other. Structural equation modelling revealed different direct and indirect influences on either visual or verbal engagement in synchronous online higher education courses. Due to the novelty of the research scope, results of this study provide a foundation for further investigation.

## Introduction

Throughout the ages, technological devices have impacted and shaped education. In particular, the digital revolution provided immense and novel opportunities within a very short period of time (Kitchin, 2014), essentially opening the door to online education.

Online learning relies on educational technology – for example, text-based tools, knowledge organisation and sharing tools, as well as website creation tools (classification as per Bower, 2016, 2017) – that allows learners to engage asynchronously with both content and peers, and at their own pace and convenience. However, direct audiovisual interaction – “prepared live interaction” (Rapanta et al., 2020, p. 935) – amongst learners, their peers, and instructors predominantly occurs in online learning settings via synchronously organized meetings, conducted through video conferencing applications.

Videoconferencing (Al-Samarraie, 2019) was established to allow learners and instructors from different places to visually and verbally participate in interactive and synchronous higher education courses while seeing each other and receiving direct verbal feedback. Different tools can be used for video conferencing that enable students and educators to interact with each other (Correia et al., 2020). They enable educators and learners to interact via file sharing and live discussion of course topics. Although it is considered a helpful, affordable, and flexible tool for online learning, many lecturers still do not perceive it as suitable for lecturing (Rapanta et al., 2020). However, due to the novelty of the situation of comprehensive synchronous online learning, there is very limited evidence on how students participate and actively engage in such courses and to what extent they are influenced by technological devices. Earlier research on students’ visual participation and engagement via webcam use is predominantly restricted to language learning, learning situations with a dyadic character, or qualitative case studies (Gillies, 2008; Kozar, 2016; Wang, 2004). However, the boost that online teaching and learning formats received in 2020 will probably have tremendous consequences for higher education in the future.

### Synchronous online sessions

Technology-based communication, interaction, and collaboration differ from teacher-student and student–student interaction in the physical classroom (An & Frick, 2006; Platt et al., 2014; Rapanta et al., 2020). For example, verbal communication during webconferencing can be more uni-directional, side conversations are not possible (except written conversation via chat), and the threshold to talk to a group of unknown people might be perceived to be different than that of on-site courses (Autor:innengruppe AEDiL, 2021; McBrien et al., 2009; Ng, 2007). Furthermore, participants can usually decide whether to be visually present via webcam.

Most previous studies on video conferencing in education focused on perceptions of video conferencing in general but did not investigate webcam use specifically (Candarli & Yuksel, 2012; Lawson et al., 2010), which is also true of studies published during the Covid-19 pandemic (Fatani, 2020). The importance of the topic, however, is illustrated by a study of management students (Giesbers et al., 2013). Performance results of a mandatory summer course indicated that students’ tool use including webcam was related to their final course performance. Similarly, teacher students in a qualitative study by Nilsen et al. (2013) reported that they are more involved in learning when using their webcam. Students’ non-use of their webcams also constituted a source of insecurity, helplessness, and frustration for instructors (Autor:innengruppe AEDiL, 2021).

Initial surveys regarding synchronous online sessions in higher education indicate that students usually do not use their webcam (Eng, 2020, March 28) or that they intent to do so is very low (Bui et al., 2020; Gherheș et al., 2021). In a comprehensive survey on students webcam use in higher education syncronous online courses, about half of the students refrained from actively participating in such courses via webcam; that is, they either did not at all or only rarely use their webcams (Bedenlier et al., 2021). From a pedagogical point of view, nonetheless, there are reasons why instructors may find webcam use by their students helpful (Maimaiti et al., 2021).

### The webcam as instructionally useful technology in synchronous online sessions

Chen and Chen (2015), in the premise of effective learning, maintain that learners exhibit a certain extent of attention during the learning process. Indeed, whether learning takes place online or in the traditional classroom, it is important for instructors to observe their students, gauge their level of attention, understanding, and progress (Chen, 2012). Facial expressions, hand raises, number of eyebrow raises and head poses can be used as indicators of understanding and attention during learning (Fan et al., 2016; Ross et al., 2013). Equally important is observing, understanding, and supporting emotional processes (Graesser, 2019; Lajoie et al., 2020; Loderer et al., 2020) as they influence motivation, attention, and learning (Hascher, 2010; Tyng et al., 2017). Emotional processes serve a signaling function (Schwarz, 2012), and students may indicate difficulty in understanding the learning material to instructors with facial expressions such as boredom, confusion, frustration, or anxiety (D'Mello, 2017). In contrast, a webcam that is turned off makes it difficult for the instructor to provide timely and constructive feedback (Racheva, 2018). Note that research shows that effective feedback is an essential component of learning (Johnson, 2016; Winstone & Carless, 2019). For example, without a webcam, it is impossible to provide spontaneous non-verbal behavioral feedback, which is a widely used and successful strategy in face-to-face instruction (Li et al., 2020). Feedback is also a way for instructors to demonstrate their own social presence and encourage students to increase their social presence as well (Ice et al., 2007). Indeed, students might feel more discomfort when communicating online in their higher education courses than during face-to-face communication (Young & Bruce, 2020). In enhancing social presence, researchers see a good way to improve student learning (Andel et al., 2020; Munoz et al., 2021; Richardson et al., 2017) and, in particular, to improve students’ emotional needs, engagement, interaction, and sense of communal learning (Kaplan-Rakowski, 2020; Rapanta et al., 2020; Sobaih et al., 2020; Whittle et al., 2020).

Difficulties in transferring direct observation-based experiences and skills from traditional learning settings to synchronous online learning settings are experienced by instructors and students alike (Händel et al., 2020a, 2020b; Sobaih et al., 2020). For example, instructional strategies to enhance learner engagement and knowledge acquisition play a vital role, but these strategies are usually acquired in traditional higher education classrooms and are not easily transferable (Kim et al., 2016; Long et al., 2017; Munoz et al., 2021). In traditional learning settings, instructors are supportive of students' learning in that they observe, monitor, and evaluate it. However, in video conferencing, social interactions are indirect and may even be visually obscured without the use of a webcam, suggesting that instructors and students alike need to develop new (metacognitive) strategies to ensure these (learning) functions are not compromised by muddled communication (Anthonysamy, 2021; Anthonysamy et al. 2020; Broadbent & Poon, 2015; Naujoks et al., 2021).

### Student engagement in video conferencing

In his seminal work, Rocca (2010) summarized research based on various methodologies from different disciplines to the effect that improving student engagement is an urgent desideratum for successful learning in higher education. In view of the rising number of students in online courses in higher education, Redmond et al. (2018) also emphasized the importance of bringing online engagement to the forefront of research. Because of its significance, we want to investigate webcam use with regard to student engagement in video conferencing.

In fact, engagement advanced as an important topical theme in online learning (Bond et al., 2020; Kebritchi et al., 2017; Martin et al., 2020). Munoz et al. (2021) refer to it as "the most recurring challenge towards online learning" (p. 2). This assessment supports numerous research findings demonstrating correlations of engagement with numerous positive learning outcomes (Kuh et al., 2008; Rocca, 2010; Sezer et al., 2017), particularly academic performance (Ayala & Manzano, 2018; Büchele, 2020; Dalelio, 2013; Kuh & Schneider, 2008; Oriol-Granado et al., 2017; Vizoso et al., 2018).

However, in 2019, only one year before higher education globally switched to online learning, the scoping review by Al-Samarraie (2019) identified only 13 articles related to video conferencing; most were related to dyadic or small group language learning and not explicitly to engagement. As the circumstances changed and video conferencing appeared as a standard teaching format in higher education (e.g., Bond et al., 2021; Skulmowksi & Rey, 2020), it becomes clearer still that, “there is still a notable lack of research to demonstrate the current use of videoconferencing in the higher education “ (Al-Samarraie, 2019, p. 122). Similarly, Ruthotto et al. (2020) concluded that no empirically validated model for online engagement exists, and we essentially do not know about the drivers and inhibitors of active and passive participation.

However, conceptual issues pose a major problem for a study of engagement. First, many and very different types of engagement are mentioned in the literature including academic, cognitive, intellectual, institutional, emotional, behavioral, social, and psychological engagement (Fredricks et al., 2016; Parsons & Taylor, 2011). For example, Burchfield and Sappington (1999, p. 290) defined engagement in terms of outcomes (''the number of unsolicited responses volunteered''), Astin (1984, p. 298) in terms of effort (''the amount of physical and psychological energy that the student devotes to the academic experience''), or Krause (2005, p.3) in terms of the use of endogenous and exogenous resources (''time, energy, and resources students devote to activities designed to enhance learning at university''). These divergent perspectives support Krause's (2005) criticism that engagement is a catch-all term.

## The current research

In this article, we highlight this definitional shortcoming, but we will not actively contribute to addressing it. Instead, we will operationalize engagement with two straightforwardly observable behaviors, webcam use as an operationalization of visual engagement, and verbal contributions during synchronous online courses as an operationalization of verbal engagement.

### Aims of the study

The current study aimed to provide a comprehensive overview of student engagement in higher education video conferencing courses; it combines the investigation of visual with verbal engagement. Previous research focused either on active participation in asynchronous learning formats like, for example, discussion forums, or on webcam use in dyadic or language learning settings. Hence, the current study broadens the field of research via a cross-disciplinary investigation of synchronous online higher education courses considering verbal as well as visual engagement.

Following Kahu (2013), we chose three types of questions for our research related to three phases of student engagement in higher education: (1) A state of engagement; (2) antecedents that influence it; and (3) consequences of engagement. Regarding the state of engagement, and with the exception of a few studies (Bedenlier et al., 2021; Castelli & Sarvary, 2021), little is known about the frequencies of webcam use and its general correlates in video conferencing. In particular, most of the research is either specified to the field of language education or relates to asynchronous online formats as discussion forums. Hence, the current work investigates students’ verbal and visual engagement in higher education courses from a cross-disciplinary but situation-specific perspective, i.e. we studied students’ active engagement regarding one specific course situation. Based on previous research (Nilsen et al., 2013), the study focuses on courses that are not restricted to lecture-based unidirectional session but that are of an interactive character. First, it was investigated how actively students engage in higher education video conferencing courses. Based on current research, it was expected that students hesitate to actively engage in video conferencing – visually as well as verbally.

- *H1a*: A considerable proportion of students are not visually present via webcam in video conferencing.
- *H1b*: A considerable proportion of students do not actively participate via verbal contributions in video conferencing.

Regarding the antecedents that influence webcam use, we focused on contextual factors for engagement in online courses. Current studies investigated course characteristics as group size or instructor participation as potential predictors of student engagement. For example, regarding online discussion forums, Kim (2013) and Kim et al. (2011) found that lecturer encouragement contributed to student participation in discussion forums but that large discussion forums were limited by lower levels of interactivity and less in-depth discussions. Similarly, Parks-Stamm et al. (2017) found significant interaction effects of group size and the amount of instructor participation on student engagement. Those effects might transfer to student engagement in video conferencing in higher education in the amount of verbal contributions. That is, with smaller groups, students might engage in more in-depth discussion. Furthermore, group size might also be negatively related to visual engagement via webcam. The anonymity of large groups might lead to a lower frequency of webcam use—analogous to perceiving smaller group sizes as conducive to relationships building (Akcaoglu & Lee, 2016).

In a study with seven Australian educators (Chen et al., 2020), lecturers scored relatively highly when asked whether they would like to see their students’ faces in a Blackboard Collaborate session. However, the study did indicate lower values regarding whether teachers would like to show their faces in a blackboard session. Although this study only refers to a very small sample of lecturers, there seems to be a discrepancy between how lecturers behave themselves and what they expect of their students. Earlier studies with a focus on language learning provide compelling insight on the matter: Although it is not clear whether results transfer to higher education in general, students and educators reported that they used their webcam only in the beginning and end of a session (Kozar, 2016).

Furthermore, we investigated potential factors related to active engagement in higher education video conferencing courses. Based on previous research regarding participation in online discussion groups (Kim, 2013; Parks-Stamm et al., 2017), we assumed that course size as well as the behavior of others (peers, lecturer) influence active verbal and visual engagement in video conferencing. In detail, we postulated direct effects of group size, lecturer and peer behavior, and perceived open communication (as one aspect of social presence in addition to group cohesion and affective expression) on visual and verbal engagement. In addition, we expected indirect effects; effect of group size and effect of lecturer behavior on engagement mediated via peer behavior.

- *H2a*: Active verbal and visual engagement are negatively related to group size and positively related to others’ behavior and perceived open communication.
- *H2b*: The effect of group size on active verbal and visual engagement is mediated by others’ behavior.
- *H2c*: The effect of lecturer encouragement on active verbal and visual engagement is mediated by peer behavior.

Following Kahu (2013), we also wanted to examine the consequences of engagement. Unique to our research context is the question of how webcam use influences other forms of engagement. In particular, we were interested in the extent to which visual and verbal engagement may influence each other. Research indicates that some students show a positive manifold of participation, i.e. different forms of participation are correlated (Bozkurt et al., 2020; Kahu, 2013; Sun et al., 2014). On the other hand, lurking (viewership or "passive participation") is a common behavior (Edelmann, 2013; Gerbic, 2006; Pala & Erdem, 2020; Ruthotto et al., 2020; Taylor, 2002). In addition to more intrinsic orientations, an instrumental orientation also plays a major role (Lashbrook, 2010). An instrumental orientation may focus on grades or on satisfying the instructor while only fulfilling basic requirements (Pala & Erdem, 2020). As a result, we considered it plausible that while there are students with a more intrinsic orientation that should lead to visual and verbal engagement, a substantial proportion of students are also satisfied with having demonstrated their motivation with either form of engagement.

- *H3*: Active verbal and visual engagement are only weakly correlated (Cohen, 1988).

## Method

### Procedure

The current research reports on the first measurement wave of a study during the winter term in Germany (November 2020 to February 2021). The survey was hosted online between November 19 and November 29, 2020, which corresponds to the middle of the third to the end of the fourth week of the semester. All students enrolled at one German university were invited via e-mail to participate in one of two online surveys, focusing either on online learning (the current survey) or on the compatibility of studying online with family life. All first year students were asked to participate in the current survey on online learning. Students enrolled in the second year or above were asked to select one of the two surveys in accordance with their month of birth. This study concerns the odd-numbered birth months; a second subsequent study will concern the other six months of the calendar year. Students were informed that participation would take approximately 25 min and that the topic in question would be digital learning. The online survey was carried out in the German language and administered via Unipark Questback EFS (https://ww2.unipark.de/).

### Sample

Participating students were recruited from one comprehensive German university. The participants were assured that their responses would remain confidential, all data were pseudonymized, and students were not disadvantaged due to non-participation. Informed consent of the participants was obtained by virtue of survey completion. Students participating in the survey could participate in a raffle where five tablet PCs were raffled off.

The current online survey was completed by 4,143 students. Within this sample, 284 students indicated that they would not participate in any video conferencing that semester and consequently, were not further surveyed. A further 237 students reported not having access to a webcam and ultimately, were also excluded from the analyses as those students did not have the option to switch their cameras on or off. Henceforth, the sample under investigation refers to 3,610 students. On average, students were 22.3 years old (*SD* = 4.5). Sample characteristics like gender distribution, migration background, SES, belonging faculty, and desired degree, are provided in Table 1.

**Table 1:** Sample Characteristics

Variable	Percentage of students
Gender	
Female	56.3
Male	29.5
Non-binary	0.3
Not indicated	13.9
Migration background (born outside Germany; non-German native language)	
Yes	11.8
No	87.7
Not indicated	0.5
SES (highest degree of the parents)	
School certificate	7.7
Vocational qualification	36.7
Higher education degree	44.3
PhD	10.6
Not indicated	0.7
Faculty	
Faculty of Humanities, Social Sciences, and Theology	27.7
Faculty of Sciences	12.9
Faculty of Business, Economics, and Law	20.6
Faculty of Engineering	16.5
Faculty of Medicine	11.0
Not indicated	11.4
Study level	
Bachelor	36.1
Master	26.6
State exam	32.6
Doctoral exam	2.8
Others	1.4
Not indicated	0.5

### Instruments

All survey items were presented in the German language. The online questionnaire consisted of three parts. First, students provided answers for the variables given in Table 1 regarding the sample description.

Second, students answered further questions regarding the last session of one specific course. Students were instructed to think about the “last session of their first course in the week that featured the characteristics of a synchronous, interactive video conferencing (that is, not solely a lecture).” This procedure was used to generate a situation-specific assessment of higher education students’ webcam use. It should enhance validity as students can refer their answers to one specific situation and do not need to aggregate their experience or behavior across several occasions and courses during their studies. The first course in the week was used as a target setting as students should be able to easily recall the week’s first course. Additionally, the week’s first course allowed for a less biased assessment of webcam use given that students might otherwise choose their most or least favorite course in the semester.

  When answering the respective items, students were reminded about this specific course session. Displayed in Table 2 are items used to assess course characteristics, course participant behavior, and student experiences as independent variables with visual and verbal engagement as dependent variables. Students reported on the course size (number of participating students), the visibility of their peers, and whether their lecturer encouraged them to participate via webcam. In addition, students reported on perceived open communication. All items were self-constructed items, except the scale for open communication, as one aspect of social presence that was a German translation of the 3-item subscale of the community of inquiry model (Díaz et al., 2010), sample item: “I felt comfortable participating in the course discussions” (Cronbach’s α = .85). Finally, as dependent variables, students should indicate whether they visually and verbally engaged in the course situation. Students indicated in which situations they turned their camera on and the degree to which they made verbal contributions. Regarding verbal contributions, the question did not distinguish between oral or written contributions and thus, can apply to both.

**Table 2:** Implemented Items Describing the Course Situation and Related Experiences

Variables	Answer type
Independent variables	
Number of course participants	Less than 5; 5 to 10; 11 to 20; 21 to 30; 30–49; 50 or more
Webcam behavior of different stakeholders	
Lecturer encourages to use webcams	no, it was not a topic; yes, friendly pointed it out; yes, using a webcam was mandatory
Webcam use of peers	no one; only me; few; about half; most; all
Social presence	
Open communication	6-point Likert scale: strongly disagree; disagree; rather disagree; rather agree; agree; strongly agree
Dependent variables	
Visual engagement	1: no, not at all; yes, 2: according to requirements; 3: yes, always
Verbal engagement	1: passive listener; 2: contributed a few times; 3: actively discussed or held a scheduled presentation

In addition, the subsample of students who indicated that their course offered breakout rooms should indicate their webcam use in this setting. Finally, students indicating that they self-organize virtual learning groups, also provided information on webcam use in this setting (again, both items with the three answer options 1: no, not at all; yes, 2: according to requirements; 3: yes, always).

### Data analysis and missing values

To investigate H1a and H1b (active engagement during video conferencing), frequencies of webcam use and verbal engagement in the course setting are reported. In addition, regarding H1a, that is, visual engagement in higher education course settings via webcam, Wilcoxon matched pairs tests were performed to compare the frequencies of student webcam use with those in breakout sessions and self-organized learning groups.

To investigate H2a through H2c, that is, potential factors directly and indirectly related to active engagement in higher education video conferencing courses, structural equation modeling (SEM) in the statistical software R (package lavaan; Rosseel, 2012) was performed. Course size (number of participants), lecturer encouragement of webcam use, and webcam use of peers were modeled as manifest categorical variables while open communication was modelled as a latent factor. The two endogenous (dependent) variables are represented by manifest categorical variables (DWLS estimator). We regressed webcam use and verbal contributions on course size, behaviors of others (peers, lecturer), and open communication. Furthermore, we modelled four indirect paths of group size and lecturer encouragement on active visual and verbal engagement, each mediated via peer behavior. To investigate H3, we examined the correlation (Spearman's rho) between visual and verbal engagement.

The data set regarding the variables of interest contained only very few missing values regarding dependent or independent variables (8 to 15 missing values per variable, which corresponds to < 0.4%). A missing values analysis regarding communication as continuous variables indicated that Little’s (1988) test of missing completely at random (MCAR) was not significant, Χ^2^(7) = 7.83, *p* = 0.35. Checking for outliers resulted in no significant outliers with *z*-scores higher than 3.29.

## Results

Table 3 reports descriptive statistics regarding the independent variables. The course in question was usually a medium to large course. There was variance regarding how many students used their webcams or whether the lecturer encouraged webcam use.

**Table 3:** Course Characteristics and Technical Settings

Variable and answer options	Percentage of students [%]
Number of course participants	
< 5	0.7
5 to 10	5.2
11 to 20	19.9
21 to 30	20.2
31 to 50	15.3
> 50	38.8
Lecturer encouragement	
Not discussed	37.1
Friendly pointed it out	54.1
Using a webcam was mandatory	8.8
Participants with webcam use	
Nobody	17.4
only me	0.2
Few	37.5
about half	14.1
Most	20.7
All	10.1

Results regarding H1a, that is, the proportion of students who used their webcam in the specific course session resulted in a relatively equal share between students who did not turn on their webcam at all (30.9%), who turned their webcam on according to requirements (36.0%) or the whole course time (33.1%). That is, about a third of the students did not turn on their webcam at all, which is in line with H1a.

To better align the results of webcam use in video conferencing, we compared student webcam use in the course setting to that of breakout rooms or self-organized learning groups for the subgroup of students who reported participation in breakout rooms or self-organized study groups. Results are given in Table 4. Wilcoxon matched pairs tests indicated significant and medium differences between the proportion of students who use their webcams in the course setting and those that use their webcams during breakout rooms, with a higher proportion of students relying on their webcam in breakout rooms. Similarly, and with a large effect size, more students use their webcam in self-organized study groups when compared to webcam use in the course setting.

**Table 4:** Webcam Use Frequency [%] Within Different Settings

	Students participating in breakout rooms		Students self-organized learning groups	
Webcam use	Course setting	Breakout room	Course setting	Study groups
*N*	1,918		1,567	
Not at all	19.0	13.0	27.8	6.2
As needed	44.5	29.5	39.1	26.7
Whole time	36.5	57.2	33.1	67.1
Wilcoxon matched pairs rank tests	*z* = 14.66, *p* < .001, *r* = .33		*z* = 21.10, *p* < .001, *r* = .53	

Regarding verbal engagement (H1b), the results indicate that only 10.5% of students actively engaged in discussions, 41.3% contributed a few times, and 48.3% of students were only passive listeners.

To investigate H2a, we calculated mean scores of all variables as well as correlations among them; see Table 5. Students’ visual and verbal engagement showed small to high correlations with the proposed set of course characteristics. In addition, as shown in Table 5, a negative and moderate correlation is identified between course size and lecturer encouragement. In addition, peer webcam use is highly correlated with the latter two.

**Table 5:** Correlation Matrix (Spearman’s rho) for the Study Variables

Variable	*Md*	2	3	4	5	6
1 Course size	5 [31–50 students]	–.31	–.54	–.08	–.48	–.36
2 Lecturer encouragement	2 [friendly pointed it out]		.60	–.02	.55	.24
3 Peers’ webcam use	3 [few students]			.10	.73	.37
4 Open communication	*M* = 3.83, *SD* = 1.02				.14	.34
5 Visual engagement (webcam use)	2 [according to requirements]					.42
6 Verbal engagement	2 [contributed a few times]					

All correlation coefficients are significant at *p* < .001 (except the correlation between lecturer’s encouragement and open communication, which is not significant)

**Fig. 1:**
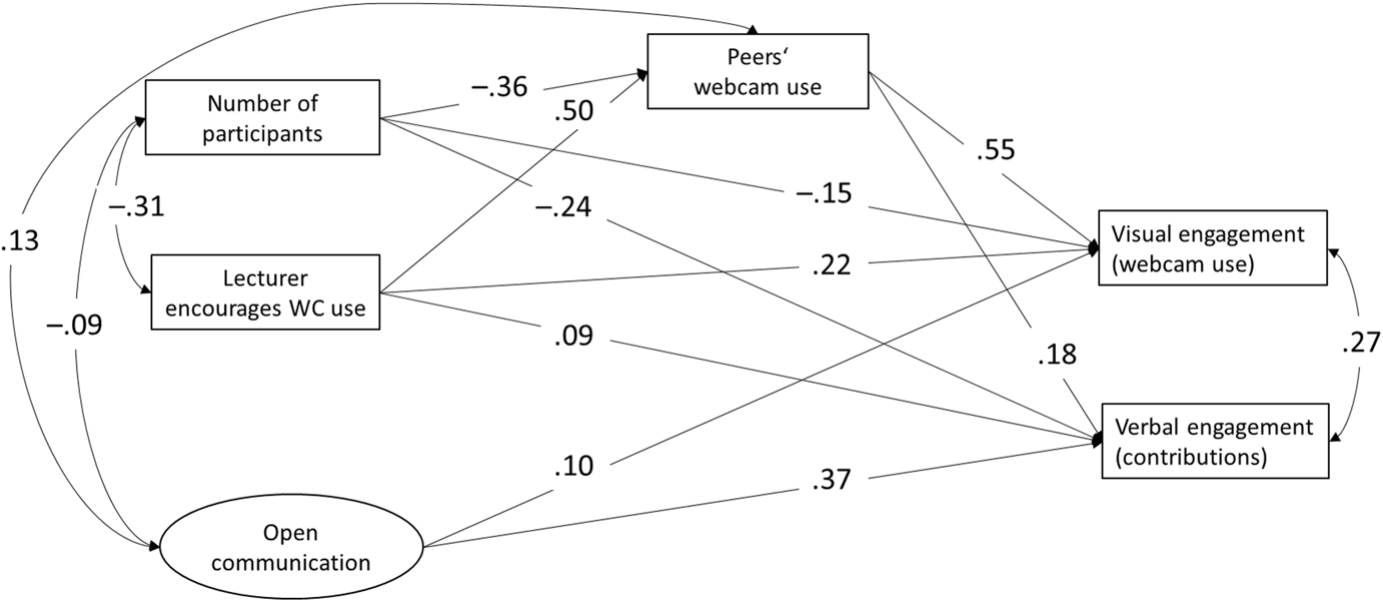
Path Analysis Model of Associations between Course-Related Variables and Active Visual and Verbal Engagement*. Note*. Coefficients presented are standardized linear regression coefficients of the final model including only significant paths. All coefficients are significant at *p* < .001

To answer H2b-c, a path model including direct and indirect effects was calculated. According to Hu and Bentler (1999), the fit of the model was very good, χ^2^(10) = 24.50, CFI = .999, TLI = .996, RMSEA = .020, SRMR = .015. Figure 1 shows the path model including all significant paths and standardized regression coefficients. Direct small effects of course size and lecturer encouragement were found on visual and verbal engagement. That is, within a smaller course size and in courses where the lecturer encouraged students to be visually present, students were visually as well as verbally more involved. Webcam use by other course participants (peers) showed a small (verbal engagement) to large (visual engagement) relationship with student active engagement. That is, when more peers were using their webcam, students engaged more in webcam use as well as in verbal communication. Finally, positive small (visual engagement) to medium (verbal engagement) effects of perceived open communication on student engagement were found. Students who perceived higher levels of open communication were more actively involved in the course situation.

Moreover, all indirect effects were of significance. That is, the paths of course size and lecturer encouragement were significantly mediated through webcam use of peers. An investigation of indirect effects showed that the relationship of the number of participants with visual and verbal engagement was mediated through webcam use of course participants (visual: β = –.20, S.E. = 0.01; verbal: β = –.06, S.E. = 0.01). In addition, the relationship between lecturer encouragement and visual/verbal engagement was mediated through webcam use of course participants (visual: β = .27, S.E. = 0.02; verbal: β = .09, S.E. = 0.02).

Indeed, especially regarding visual engagement, peer webcam use played an important moderating role while the direct effect of course size and lecturer encouragement was small. In contrast, for verbal engagement, perceived open communication seemed more important for student active engagement.

Finally, in investigating H3 we looked at the correlation between visual and verbal engagement. Spearman's rho was only .42, thus the resulting effect size represents a relatively weak association.

## Discussion

Scholars agree that new technologies have already changed higher education and will continue to do so (Altbach et al., 2009; Isaías, 2018; Marinagi et al., 2013). Kebritchi et al. (2017) start their literature review by stating that „online education changes *all* components of teaching and learning in higher education “ (p. 4; emphasis added). Various scholars even noted an irreversible trend wherein higher education is progressively displaced from the traditional classroom and instead continues to evolve in online settings (Isaías, 2018). This trend was accelerated by the pandemic in 2020, when higher education worldwide switched to online learning, employing a mix of asynchronous and synchronous online teaching formats. Our empirical study focused on the impact of webcam use on higher education online courses and, in particular, on student engagement. In particular, we think the use of a webcam in video conferencing plays a significantly large role in student engagement and creates an important area of applied research.

There is practically no research on the (non)use of webcams and their influence on synchronous online courses (exceptions are Bedenlier et al., 2021; Castelli & Sarvary, 2021). It is therefore not yet clear whether a webcam will have the potential to become a "disruptive technology" (Flavin, 2012), a "disruptive innovation" (Christensen & Raynor, 2003), or a sustaining technology in higher education (Christensen & Eyring, 2011). The latter would certainly have the potential to enhance existing online teaching practices. However, we maintain that a webcam also has the potential to be a disruptive technology, as it literally disrupts established teaching practices, such as interactions with students, instructor feedback, student engagement, etc.

The present study used a cross-disciplinary situation-specific approach to survey student engagement in online higher education courses in a large sample of students at a comprehensive German university. Due to the increasing amount of synchronous higher education courses at present but also in the future (Garrison et al., 2010), this is important work as it informs on potential characteristics that contribute to student engagement in such settings.

Students were asked to describe their visual engagement via webcam and their verbal engagement via contribution in discussions in the last session of one specific course. To avoid a conflict wherein students select a ‘preferred’ course, the specific course in question was not self-selected but the first course in the students’ schedules of the respective week. Such a situation-specific approach was chosen to lend validity to results. For example, when students differ in webcam use behavior due to different courses, their answers might be biased due to an aggregate perspective, or students might experience difficulties in answering the questions because the point of reference is not clear.

In line with current research (Bedenlier et al., 2021), the study found that about a third of students hesitated to be visually present in video conferencing via webcam and a further third of students used their webcams due to course requirements. Notably, these numbers refer only to those students that participated in the video conferencing, that is, who were part of the respective session. Moreover, there might be a group of students that would fail to enter the meeting room at all. Interestingly, more students made use of the webcam function in breakout rooms or self-organized synchronous online sessions. Accordingly, the installation of breakout rooms by lecturers (Pisutova et al., 2018; Reinholz et al., 2020) might lower the inhibition threshold of being visually present.

Furthermore, nearly half of the participating students only passively participated in the course session. As visual and verbal engagement were only weakly correlated, there was some tendency among students to either make use of both visual and verbal ways to participate in video conferencing courses.

Results of the SEM confirmed our hypotheses. Students were more engaged in a video conferencing session if the course comprised fewer participants, and if the lecturer encouraged them to be visually present. That is, previous results regarding student engagement in asynchronous online education (Kim, 2013; Parks-Stamm et al., 2017; Ruthotto et al., 2020) seem to transfer to video conferencing. For visual engagement via webcam, peer behavior was an important factor—directly and as a mediator for the relationship of course size and lecturer encouragement. In contrast, for verbal engagement, open communication was most relevant. That is, students who experience that they can communicate openly and feel comfortable in the online course communication platform are those that engage more in course discussions (Bedenlier et al., 2021).

Regarding the hypotheses of our study, our findings have implications for higher education that are worth considering. With H1 and H2, we had assumed that a considerable proportion of students is neither visually present via webcam, nor do they actively participate via verbal contributions in video conferencing. In fact, often an instructor and students are “talking into a void” (O'Conaill et al., 1993), which can have negative emotional, motivational, and social consequences (Butz et al., 2015; Händel et al., 2020a, 2020b). Without a webcam switched on, many advantages of synchronous online learning such as opportunities for higher interactivity, timely and constructive feedback, real‐time collaborative learning (Racheva, 2018), and the forging of a stronger sense of community that fosters interactions (Lin & Gao, 2020) can only be utilized to a limited extent. Considering the potential advantages of students having their cameras on and the disadvantages of having their cameras off (Castelli & Sarvary, 2021), it seems extremely important to consider strategies to encourage students to turn on their webcams and verbally participate. However, our finding on H3 shows that webcam use does not automatically lead to verbal engagement and verbal engagement does not automatically imply webcam use.

Suggestions as to how students can be encouraged to better participate in online courses are outlined by our findings on H2. These show that reasons given for keeping the webcam turned off are manifold and go beyond general concerns regarding technical equipment (Händel et al., 2020a; Naveh & Shelef, 2021) or privacy concerns (Castelli & Sarvary, 2021; Rajab & Soheib, 2021; Sobaih et al., 2020). To achieve high student engagement in video conferencing, interactive courses should be of a rather small size. That is, while online lecture-based teaching formats might have the advantage of no longer being limited by room capacities, higher education courses that aim to be of an interactive nature should limit participant size—or alternatively, should allow for smaller subgroup discussions in breakout rooms (Reinholz et al., 2020). Those environmental characteristics should lead to higher use of webcams as well as more in-depth and interactive discussions. Aside from the observation that lecturers encourage or discourage student engagement, educators need to take care to establish a group atmosphere where students experience open communication as one component of social presence. Another factor was the observation of peer behavior. Educators could, for example, choose teaching formats in which more students feel comfortable enough to switch on their cameras. Our study shows that break-out rooms can be a helpful modification to the teaching format.

However, it is also important to consider that encouraging students to use their webcams has further implications. Encouragement may easily turn into pressure and, for example, trigger privacy concerns among students (Castelli & Sarvary, 2021; Rajab & Soheib, 2021; Sobaih et al., 2020). Consequently, various higher education institutions have already formulated recommendations for online participation that also include its related ethical aspects (Harvard University, 2021; Stanford University, 2021).

### Limitations and future prospects

In this section, we identify four specific limitations of the empirical study we presented. However, we would also like to constructively point out further limitations, the consideration of which is important for future research.

The presented findings refer to cross-sectional data of an online survey. Our study is strengthened both by our approach and by circumstance, particularly in the momentary situation of nearly exhaustive online education. Still, this leads to several limitations.

First, the sample is a non-randomized sample. The online survey probably had attracted specific groups of students (Wright, 2005). For example, students who are especially interested in or who are annoyed by online education might have a higher motivation to participate in the surveys. Furthermore, due to design reasons, all students lacking adequate equipment were excluded from further data analyses. Those sample constraints need to be considered carefully as they might limit the validity of the study results.

Second, all obtained results are based on self-reports and thus, might be biased. Student answers were directed to a concrete situation in order to avoid bias due to aggregation of experiences across courses or sessions. Still, we did not track students’ actual webcam usage times, number of verbal contributions, or even the quality of their active engagement (Hrastinski, 2008; Vonderwell & Zachariah, 2005). That is, similarly to discussion forums, it is argued that engagement is more than the total number of student verbal contributions. In accordance, self-reports (and single items) might have implications for the reliability of results.

Third, the influence of course size or lecturer encouragement was not manipulated experimentally. For example, many students derived their answers from experience in large courses where it is—by nature of the setting—less possible or necessary to participate verbally and visually. In these large course contexts, it is often the case that a limited number of students are visible on the computer screen. Moreover, within the regular course duration of 90 min, and when compared to smaller settings, a verbal contribution is less probable in courses with many students. Still, results were in line with research on online discussion forums (Kim, 2013; Parks-Stamm et al., 2017) comparing small groups with less than 15 students and medium groups with 15–30 students. That is, the current study found significant relationships between group size and student active participation.

Fourth, due to the cross-sectional nature of the analysis, the path coefficients do not necessarily inform on causal relationships between the variables. For example, while our hypotheses directly assessed the influence of course variables on student course engagement, it is also plausible that students who do not want to be visible via webcam explicitly choose courses with more participants where they know that it is easier to “hide.” Similarly, students might perceive higher open communication in a course situation because many students engaged in verbal discussions. Hence, to reproduce and further enrich our research findings, future studies should consider those aspects in their study designs.

Lastly, it is important to consider another limitation as far as it concerns future studies on webcam use in online courses in higher education. Like many models assessing the acceptance and impact of new technologies, we have focused on the evaluation of a single group, usually referred to as end-users (Davis, 1989; Venkatesh et al., 2003). However, many other stakeholders are involved in online learning settings, so an ecosystem perspective that considers other actors (for example, instructor, university leadership; Rapanta et al., 2020) and contexts (for example, ethical; de Souza Rodrigues et al., 2021) is beneficial.

Specifically regarding the study of engagement, we would like to point out three aspects. First, only two indicators of engagement were considered: Visual and verbal engagement. Of course, a finer distinction can and must be eventually made here. For example, Moubayed et al. (2020) proposed 12 engagement metrics divided in the sub-categories: interaction-related and effort-related. Other authors point out that in addition to behavioral forms, other types of engagement should be examined such as emotional and cognitive engagement (Fredricks et al., 2016). Second, future research should also move toward distinguishing degrees of engagement. Quaye et al. (2019) point out that there is a qualitative difference between involvement and engagement, for example. One can be involved in something without being engaged. Third, it is a necessary next step to investigate under which circumstances visual and/or verbal engagement is beneficial—e.g., do motivational variables, collaboration in the course, or performance results (Giesbers et al., 2013; Wekerle et al., 2020) make a difference.

Finally, we would like to return to our opening statement that throughout the ages, technological devices have impacted and shaped education. In our study, we found preliminary evidence suggesting that webcam use could also have such an influence. However, the magnitude of this influence, the exact areas of higher education it affects, and the exact psychological mechanisms involved cannot be determined at this time. We look forward to research that addresses these questions.
